# *N*′-(3,5-Dibromo-2-hy­droxy­benzyl­idene)-2-methyl­benzohydrazide

**DOI:** 10.1107/S1600536810048889

**Published:** 2010-11-30

**Authors:** Chun-Bao Tang

**Affiliations:** a Department of Chemistry, Jiaying University, Meizhou 514015, People’s Republic of China

## Abstract

The asymmetric unit of the title compound, C_15_H_12_Br_2_N_2_O_2_, contains two independent mol­ecules in which the dihedral angles between the benzene rings are 49.5 (7) and 66.4 (7)°. Intra­molecular O—H⋯N hydrogen bonds generate *S*(6) ring motifs in each mol­ecule. In the crystal, mol­ecules are linked through inter­molecular N—H⋯O hydrogen bonds, forming chains along the *b* axis.

## Related literature

For general background to hydrazones, see: Rasras *et al.* (2010 ▶); Pyta *et al.* (2010 ▶); Angelusiu *et al.* (2010 ▶). For related structures, see: Fun *et al.* (2008 ▶); Singh & Singh (2010 ▶); Ahmad *et al.* (2010 ▶); Tang (2010 ▶). For reference bond-length data, see: Allen *et al.* (1987 ▶) and for hydrogen-bond motifs, see: Bernstein *et al.* (1995 ▶).
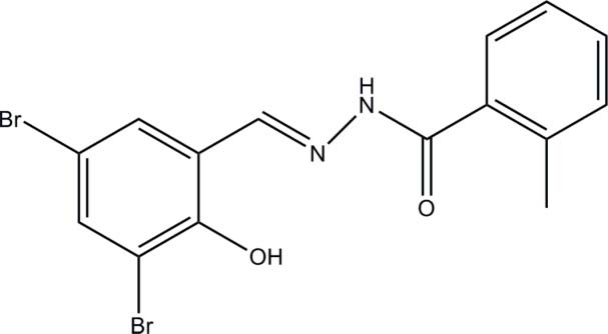


## Experimental

### 

#### Crystal data

C_15_H_12_Br_2_N_2_O_2_
*M* *_r_* = 412.09
Monoclinic, 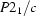

*a* = 18.636 (3) Å
*b* = 9.606 (2) Å
*c* = 19.943 (3) Å
β = 113.726 (2)°
*V* = 3268.4 (10) Å^3^
*Z* = 8
Mo *K*α radiation
μ = 4.97 mm^−1^
*T* = 298 K
0.17 × 0.13 × 0.12 mm

#### Data collection

Bruker SMART CCD area-detector diffractometer
Absorption correction: multi-scan (*SADABS*; Sheldrick, 1996 ▶) *T*_min_ = 0.486, *T*_max_ = 0.587
17117 measured reflections
6973 independent reflections
2208 reflections with *I* > 2σ(*I*)
*R*_int_ = 0.116

#### Refinement

*R*[*F* ^2^ > 2σ(*F* ^2^)] = 0.053
*wR*(*F* ^2^) = 0.159
*S* = 0.89
6973 reflections
389 parameters
2 restraints
H atoms treated by a mixture of independent and constrained refinement
Δρ_max_ = 0.32 e Å^−3^
Δρ_min_ = −0.49 e Å^−3^

### 

Data collection: *SMART* (Bruker, 2002 ▶); cell refinement: *SAINT* (Bruker, 2002 ▶); data reduction: *SAINT*; program(s) used to solve structure: *SHELXS97* (Sheldrick, 2008 ▶); program(s) used to refine structure: *SHELXL97* (Sheldrick, 2008 ▶); molecular graphics: *SHELXTL* (Sheldrick, 2008 ▶); software used to prepare material for publication: *SHELXL97*.

## Supplementary Material

Crystal structure: contains datablocks global, I. DOI: 10.1107/S1600536810048889/sj5065sup1.cif

Structure factors: contains datablocks I. DOI: 10.1107/S1600536810048889/sj5065Isup2.hkl

Additional supplementary materials:  crystallographic information; 3D view; checkCIF report

## Figures and Tables

**Table 1:** Hydrogen-bond geometry (Å, °)

*D*—H⋯*A*	*D*—H	H⋯*A*	*D*⋯*A*	*D*—H⋯*A*
N2—H2⋯O4^i^	0.90 (1)	1.85 (2)	2.743 (8)	172 (7)
N4—H4⋯O2	0.90 (1)	1.92 (2)	2.815 (8)	174 (7)
O3—H3⋯N3	0.82	1.90	2.624 (8)	146
O1—H1⋯N1	0.82	1.87	2.584 (8)	145

Symmetry code: (i) 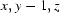.

**Fig. 1.**
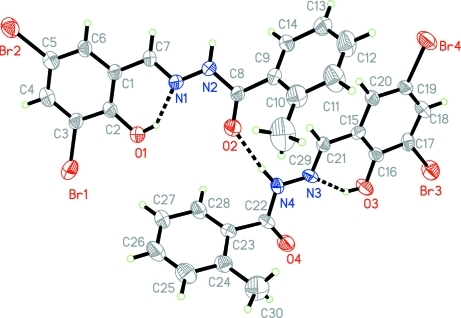
The molecular structure of the compound, showing the atom-numbering scheme. Displacement ellipsoids are drawn at the 30% probability level. Hydrogen atoms are shown as spheres of arbitrary radius.

**Fig. 2.**
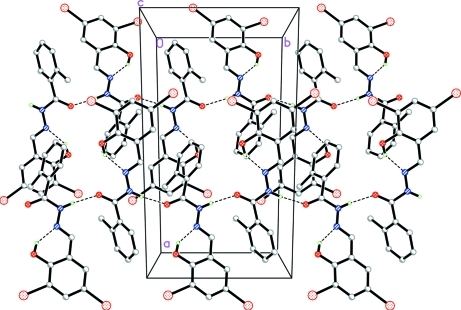
Molecular packing of the title compound, with hydrogen bonds shown as dashed lines.

## supplementary crystallographic information

### Comment

Hydrazone compounds have received much attention in biological and structural chemistry in the last few years (Rasras *et al.*, 2010; Pyta *et al.*, 2010; Angelusiu *et al.*, 2010; Fun *et al.*, 2008; Singh & Singh, 2010; Ahmad *et al.*, 2010). In the present paper, the author reports the crystal structure of the new title hydrazone compound (Fig. 1).

The asymmetric unit of the title compound contains two independent molecules. The dihedral angles between the two benzene rings in the two molecules are 49.5 (7) and 66.4 (7)°, respectively. The torsion angles C1—C7—N1—N2, C7—N1—N2—C8, N1—N2—C8—C9, C15—C21—N3—N4, C21—N3—N4—C22, and N3—N4—C22—C23 are 3.9 (6), 4.3 (6), 1.8 (6), 2.4 (6), 7.3 (6), and 2.4 (6)°, respectively.  Bond lengths in the molecules are normal (Allen *et al.*, 1987) and comparable to those in the similar compound the author reported recently (Tang, 2010). Intramolecular O1—H1···N1 and O3—H3···N3 hydrogen bonds generate S(6) ring motifs in each molecule (Bernstein *et al.*, 1995). In the crystal structure, molecules are linked through intermolecular N—H···O hydrogen bonds (Table 1), forming chains along the *b* axis (Fig. 2).

### Experimental

3,5-Dibromo-2-hydroxybenzaldehyde (0.1 mmol, 28.0 mg) and 2-methylbenzohydrazide (0.1 mmol, 15.0 mg) were dissolved in methanol (20 ml). The mixture was stirred at reflux for 10 min to give a clear colourless solution. Colourless block-shaped crystals of the compound were formed by slow evaporation of the solvent over several days.

### Refinement

The amino H atoms were located in a difference Fourier map and refined isotropically, with the N—H distances restrained to 0.90 (1) Å [*U*_iso_(H) = 0.08 Å^2^]. Other H atoms were constrained to ideal geometries and refined as riding, with Csp^2^—H = 0.93 Å, C(methyl)—H = 0.96 Å, and O—H = 0.82 Å; *U*_iso_(H) = 1.2*U*_eq_(C) and 1.5*U*_eq_(O and C_methyl_).

### Crystal data

**Table:**

C_15_H_12_Br_2_N_2_O_2_	*F*(000) = 1616
*M**_r_* = 412.09	*D*_x_ = 1.675 Mg m^−^^3^
Monoclinic, *P*2_1_/*c*	Mo *K*α radiation, λ = 0.71073 Å
Hall symbol: -P 2ybc	Cell parameters from 1348 reflections
*a* = 18.636 (3) Å	θ = 2.5–24.1°
*b* = 9.606 (2) Å	µ = 4.97 mm^−^^1^
*c* = 19.943 (3) Å	*T* = 298 K
β = 113.726 (2)°	Block, colourless
*V* = 3268.4 (10) Å^3^	0.17 × 0.13 × 0.12 mm
*Z* = 8	

### Data collection

**Table:**

Bruker SMART CCD area-detector diffractometer	6973 independent reflections
Radiation source: fine-focus sealed tube	2208 reflections with *I* > 2σ(*I*)
graphite	*R*_int_ = 0.116
ω scans	θ_max_ = 27.0°, θ_min_ = 2.2°
Absorption correction: multi-scan (*SADABS*; Sheldrick, 1996)	*h* = −23→19
*T*_min_ = 0.486, *T*_max_ = 0.587	*k* = −12→11
17117 measured reflections	*l* = −25→25

### Refinement

**Table:**

Refinement on *F*^2^	Primary atom site location: structure-invariant direct methods
Least-squares matrix: full	Secondary atom site location: difference Fourier map
*R*[*F*^2^ > 2σ(*F*^2^)] = 0.053	Hydrogen site location: inferred from neighbouring sites
*wR*(*F*^2^) = 0.159	H atoms treated by a mixture of independent and constrained refinement
*S* = 0.89	*w* = 1/[σ^2^(*F*_o_^2^)] where *P* = (*F*_o_^2^ + 2*F*_c_^2^)/3
6973 reflections	(Δ/σ)_max_ < 0.001
389 parameters	Δρ_max_ = 0.32 e Å^−^^3^
2 restraints	Δρ_min_ = −0.49 e Å^−^^3^

### Special details

**Table:**

Geometry. All esds (except the esd in the dihedral angle between two l.s. planes) are estimated using the full covariance matrix. The cell esds are taken into account individually in the estimation of esds in distances, angles and torsion angles; correlations between esds in cell parameters are only used when they are defined by crystal symmetry. An approximate (isotropic) treatment of cell esds is used for estimating esds involving l.s. planes.
Refinement. Refinement of F^2^ against ALL reflections. The weighted R-factor wR and goodness of fit S are based on F^2^, conventional R-factors R are based on F, with F set to zero for negative F^2^. The threshold expression of F^2^ > 2sigma(F^2^) is used only for calculating R-factors(gt) etc. and is not relevant to the choice of reflections for refinement. R-factors based on F^2^ are statistically about twice as large as those based on F, and R- factors based on ALL data will be even larger.

### Fractional atomic coordinates and isotropic or equivalent isotropic displacement parameters (Å^2^)

**Table:**

	*x*	*y*	*z*	*U*_iso_*/*U*_eq_	
Br1	0.69344 (6)	0.40876 (10)	0.38431 (6)	0.0971 (4)	
Br2	0.72350 (5)	−0.17458 (10)	0.37439 (6)	0.0928 (4)	
Br3	−0.06953 (6)	0.85420 (10)	−0.01006 (5)	0.0876 (4)	
Br4	−0.12081 (6)	0.29585 (11)	0.04659 (6)	0.0984 (4)	
N1	0.4069 (4)	0.1700 (7)	0.3119 (3)	0.0615 (18)	
N2	0.3301 (4)	0.1455 (7)	0.3007 (4)	0.0614 (19)	
N3	0.1898 (4)	0.6601 (6)	0.2214 (3)	0.0576 (18)	
N4	0.2608 (4)	0.6440 (7)	0.2795 (4)	0.0605 (18)	
O1	0.5235 (3)	0.3360 (6)	0.3345 (3)	0.0757 (17)	
H1	0.4775	0.3154	0.3240	0.114*	
O2	0.3146 (3)	0.3703 (6)	0.3204 (3)	0.0781 (18)	
O3	0.0844 (3)	0.8048 (6)	0.1160 (3)	0.0726 (16)	
H3	0.1266	0.7907	0.1504	0.109*	
O4	0.2768 (3)	0.8762 (6)	0.2838 (3)	0.0822 (19)	
C1	0.5314 (4)	0.0854 (9)	0.3282 (4)	0.051 (2)	
C2	0.5659 (5)	0.2190 (9)	0.3415 (4)	0.057 (2)	
C3	0.6469 (5)	0.2318 (8)	0.3630 (4)	0.060 (2)	
C4	0.6930 (5)	0.1155 (10)	0.3713 (4)	0.067 (2)	
H4A	0.7467	0.1249	0.3851	0.081*	
C5	0.6590 (5)	−0.0157 (9)	0.3591 (4)	0.065 (2)	
C6	0.5792 (5)	−0.0284 (9)	0.3376 (4)	0.058 (2)	
H6	0.5569	−0.1168	0.3292	0.069*	
C7	0.4486 (4)	0.0642 (9)	0.3118 (4)	0.057 (2)	
H7	0.4265	−0.0242	0.3015	0.069*	
C8	0.2854 (5)	0.2545 (10)	0.3053 (4)	0.055 (2)	
C9	0.2028 (5)	0.2208 (9)	0.2904 (5)	0.061 (2)	
C10	0.1668 (6)	0.2838 (11)	0.3318 (5)	0.088 (3)	
C11	0.0869 (7)	0.2519 (13)	0.3119 (7)	0.109 (4)	
H11	0.0607	0.2906	0.3384	0.131*	
C12	0.0470 (7)	0.1646 (14)	0.2540 (7)	0.111 (4)	
H12	−0.0059	0.1473	0.2417	0.133*	
C13	0.0832 (6)	0.1029 (9)	0.2144 (6)	0.089 (3)	
H13	0.0561	0.0433	0.1757	0.107*	
C14	0.1627 (5)	0.1327 (9)	0.2342 (5)	0.073 (3)	
H14	0.1888	0.0911	0.2083	0.087*	
C15	0.0682 (4)	0.5631 (8)	0.1399 (4)	0.048 (2)	
C16	0.0418 (5)	0.6869 (9)	0.1003 (4)	0.054 (2)	
C17	−0.0330 (5)	0.6879 (9)	0.0439 (4)	0.059 (2)	
C18	−0.0814 (5)	0.5726 (9)	0.0281 (4)	0.066 (2)	
H18	−0.1315	0.5755	−0.0090	0.079*	
C19	−0.0538 (4)	0.4536 (8)	0.0683 (4)	0.057 (2)	
C20	0.0207 (4)	0.4455 (9)	0.1218 (4)	0.056 (2)	
H20	0.0394	0.3616	0.1458	0.067*	
C21	0.1458 (4)	0.5550 (8)	0.1993 (4)	0.051 (2)	
H21	0.1636	0.4695	0.2217	0.061*	
C22	0.3020 (5)	0.7621 (9)	0.3066 (4)	0.051 (2)	
C23	0.3807 (5)	0.7368 (8)	0.3682 (4)	0.051 (2)	
C24	0.4012 (5)	0.8118 (9)	0.4335 (5)	0.065 (2)	
C25	0.4759 (7)	0.7929 (11)	0.4871 (5)	0.103 (3)	
H25	0.4908	0.8405	0.5312	0.124*	
C26	0.5285 (6)	0.7049 (11)	0.4762 (6)	0.102 (3)	
H26	0.5786	0.6952	0.5129	0.122*	
C27	0.5087 (5)	0.6310 (10)	0.4121 (6)	0.102 (3)	
H27	0.5443	0.5710	0.4052	0.122*	
C28	0.4339 (5)	0.6489 (9)	0.3583 (5)	0.075 (3)	
H28	0.4193	0.6003	0.3145	0.090*	
C29	0.2102 (6)	0.3762 (12)	0.3981 (6)	0.138 (5)	
H29A	0.2560	0.3286	0.4313	0.208*	
H29B	0.1763	0.3970	0.4226	0.208*	
H29C	0.2254	0.4613	0.3822	0.208*	
C30	0.3438 (6)	0.9038 (10)	0.4487 (5)	0.111 (4)	
H30A	0.3303	0.9819	0.4159	0.167*	
H30B	0.3673	0.9365	0.4983	0.167*	
H30C	0.2974	0.8516	0.4415	0.167*	
H4	0.281 (4)	0.559 (3)	0.295 (4)	0.080*	
H2	0.309 (4)	0.060 (3)	0.291 (4)	0.080*	

### Atomic displacement parameters (Å^2^)

**Table:**

	*U*^11^	*U*^22^	*U*^33^	*U*^12^	*U*^13^	*U*^23^
Br1	0.0765 (7)	0.0809 (7)	0.1295 (9)	−0.0250 (6)	0.0369 (7)	−0.0015 (7)
Br2	0.0685 (6)	0.0922 (8)	0.1017 (8)	0.0176 (6)	0.0175 (6)	−0.0253 (6)
Br3	0.0767 (7)	0.0865 (8)	0.0826 (7)	0.0029 (5)	0.0143 (6)	0.0252 (6)
Br4	0.0707 (7)	0.0878 (8)	0.1113 (8)	−0.0274 (6)	0.0101 (6)	−0.0070 (6)
N1	0.051 (5)	0.047 (4)	0.067 (5)	−0.004 (4)	0.004 (4)	0.006 (4)
N2	0.046 (5)	0.045 (5)	0.083 (5)	−0.004 (4)	0.015 (4)	0.007 (4)
N3	0.051 (4)	0.051 (4)	0.059 (5)	−0.013 (4)	0.009 (4)	−0.015 (4)
N4	0.040 (4)	0.056 (5)	0.065 (5)	0.005 (4)	0.000 (4)	0.001 (4)
O1	0.049 (3)	0.062 (4)	0.104 (5)	−0.003 (3)	0.019 (4)	0.015 (3)
O2	0.060 (4)	0.057 (4)	0.107 (5)	0.006 (3)	0.022 (4)	−0.006 (4)
O3	0.060 (4)	0.066 (4)	0.073 (4)	−0.019 (3)	0.006 (3)	0.006 (3)
O4	0.069 (4)	0.047 (4)	0.095 (5)	−0.004 (3)	−0.004 (4)	0.012 (4)
C1	0.050 (5)	0.054 (6)	0.041 (5)	−0.007 (5)	0.009 (4)	−0.006 (4)
C2	0.053 (6)	0.064 (7)	0.041 (5)	−0.002 (5)	0.005 (4)	0.005 (5)
C3	0.060 (6)	0.061 (6)	0.049 (5)	−0.013 (5)	0.010 (5)	−0.001 (4)
C4	0.053 (5)	0.091 (8)	0.055 (6)	0.006 (6)	0.018 (5)	−0.007 (6)
C5	0.062 (6)	0.073 (7)	0.056 (6)	0.010 (5)	0.019 (5)	−0.021 (5)
C6	0.050 (5)	0.063 (6)	0.050 (5)	−0.003 (5)	0.009 (4)	−0.010 (5)
C7	0.042 (5)	0.057 (6)	0.061 (5)	−0.006 (5)	0.009 (4)	0.001 (5)
C8	0.046 (6)	0.053 (6)	0.047 (5)	−0.010 (5)	0.000 (4)	0.006 (5)
C9	0.048 (6)	0.054 (6)	0.075 (7)	−0.011 (5)	0.019 (5)	0.001 (5)
C10	0.087 (8)	0.112 (9)	0.071 (7)	−0.003 (7)	0.038 (7)	0.017 (6)
C11	0.085 (9)	0.168 (12)	0.096 (9)	0.009 (8)	0.058 (8)	0.026 (9)
C12	0.074 (8)	0.164 (13)	0.101 (10)	−0.006 (8)	0.042 (8)	0.051 (9)
C13	0.073 (8)	0.080 (7)	0.105 (8)	−0.008 (6)	0.026 (7)	0.028 (6)
C14	0.048 (6)	0.066 (7)	0.101 (8)	0.005 (5)	0.027 (6)	0.017 (6)
C15	0.055 (5)	0.040 (5)	0.046 (5)	0.006 (4)	0.019 (4)	−0.001 (4)
C16	0.051 (5)	0.060 (6)	0.054 (6)	−0.010 (5)	0.024 (5)	−0.017 (5)
C17	0.046 (5)	0.074 (6)	0.049 (5)	0.009 (5)	0.011 (5)	0.018 (5)
C18	0.052 (5)	0.082 (7)	0.060 (6)	−0.019 (6)	0.018 (5)	−0.007 (5)
C19	0.050 (5)	0.050 (6)	0.057 (6)	−0.004 (4)	0.007 (5)	−0.002 (5)
C20	0.044 (5)	0.064 (6)	0.046 (5)	0.000 (5)	0.004 (4)	−0.010 (4)
C21	0.046 (5)	0.061 (6)	0.034 (5)	0.007 (4)	0.003 (4)	−0.009 (4)
C22	0.049 (5)	0.056 (6)	0.046 (5)	−0.018 (5)	0.016 (5)	−0.009 (5)
C23	0.052 (5)	0.049 (5)	0.049 (6)	−0.008 (4)	0.017 (5)	−0.005 (4)
C24	0.067 (6)	0.073 (6)	0.049 (6)	−0.002 (5)	0.017 (5)	−0.004 (5)
C25	0.104 (9)	0.116 (9)	0.055 (7)	0.005 (8)	−0.004 (7)	−0.018 (6)
C26	0.074 (8)	0.101 (9)	0.093 (9)	−0.013 (7)	−0.007 (7)	−0.013 (7)
C27	0.058 (7)	0.092 (8)	0.114 (9)	0.006 (6)	−0.008 (6)	−0.028 (7)
C28	0.058 (6)	0.078 (7)	0.070 (6)	−0.004 (5)	0.005 (6)	−0.003 (5)
C29	0.124 (10)	0.209 (14)	0.103 (9)	−0.013 (9)	0.067 (8)	−0.035 (9)
C30	0.121 (8)	0.144 (10)	0.078 (7)	0.008 (8)	0.049 (7)	−0.012 (7)

### Geometric parameters (Å, °)

**Table:**

Br1—C3	1.878 (8)	C11—H11	0.9300
Br2—C5	1.890 (8)	C12—C13	1.364 (13)
Br3—C17	1.894 (7)	C12—H12	0.9300
Br4—C19	1.900 (8)	C13—C14	1.402 (11)
N1—C7	1.280 (8)	C13—H13	0.9300
N1—N2	1.377 (8)	C14—H14	0.9300
N2—C8	1.364 (10)	C15—C20	1.391 (9)
N2—H2	0.898 (10)	C15—C16	1.402 (10)
N3—C21	1.263 (8)	C15—C21	1.457 (9)
N3—N4	1.372 (8)	C16—C17	1.396 (10)
N4—C22	1.355 (9)	C17—C18	1.382 (10)
N4—H4	0.898 (10)	C18—C19	1.371 (10)
O1—C2	1.348 (8)	C18—H18	0.9300
O1—H1	0.8200	C19—C20	1.370 (9)
O2—C8	1.221 (8)	C20—H20	0.9300
O3—C16	1.345 (8)	C21—H21	0.9300
O3—H3	0.8200	C22—C23	1.507 (10)
O4—C22	1.207 (8)	C23—C28	1.375 (10)
C1—C6	1.375 (9)	C23—C24	1.400 (10)
C1—C2	1.412 (10)	C24—C25	1.384 (11)
C1—C7	1.457 (9)	C24—C30	1.508 (11)
C2—C3	1.399 (10)	C25—C26	1.378 (12)
C3—C4	1.377 (10)	C25—H25	0.9300
C4—C5	1.387 (10)	C26—C27	1.376 (12)
C4—H4A	0.9300	C26—H26	0.9300
C5—C6	1.378 (9)	C27—C28	1.386 (11)
C6—H6	0.9300	C27—H27	0.9300
C7—H7	0.9300	C28—H28	0.9300
C8—C9	1.481 (10)	C29—H29A	0.9600
C9—C14	1.363 (10)	C29—H29B	0.9600
C9—C10	1.396 (11)	C29—H29C	0.9600
C10—C11	1.411 (13)	C30—H30A	0.9600
C10—C29	1.526 (12)	C30—H30B	0.9600
C11—C12	1.377 (14)	C30—H30C	0.9600
			
C7—N1—N2	117.1 (6)	C20—C15—C21	119.2 (7)
C8—N2—N1	118.7 (6)	C16—C15—C21	120.8 (7)
C8—N2—H2	119 (5)	O3—C16—C17	119.1 (8)
N1—N2—H2	122 (5)	O3—C16—C15	122.9 (7)
C21—N3—N4	118.3 (7)	C17—C16—C15	118.0 (8)
C22—N4—N3	116.2 (6)	C18—C17—C16	121.8 (8)
C22—N4—H4	122 (5)	C18—C17—Br3	119.6 (6)
N3—N4—H4	122 (5)	C16—C17—Br3	118.6 (7)
C2—O1—H1	109.5	C19—C18—C17	118.5 (7)
C16—O3—H3	109.5	C19—C18—H18	120.8
C6—C1—C2	118.4 (7)	C17—C18—H18	120.8
C6—C1—C7	119.3 (8)	C20—C19—C18	121.8 (7)
C2—C1—C7	122.1 (8)	C20—C19—Br4	119.9 (7)
O1—C2—C3	118.3 (8)	C18—C19—Br4	118.3 (6)
O1—C2—C1	122.3 (7)	C19—C20—C15	119.7 (7)
C3—C2—C1	119.4 (8)	C19—C20—H20	120.2
C4—C3—C2	120.6 (8)	C15—C20—H20	120.2
C4—C3—Br1	119.9 (7)	N3—C21—C15	122.2 (7)
C2—C3—Br1	119.5 (7)	N3—C21—H21	118.9
C3—C4—C5	119.8 (8)	C15—C21—H21	118.9
C3—C4—H4A	120.1	O4—C22—N4	122.5 (8)
C5—C4—H4A	120.1	O4—C22—C23	123.9 (7)
C6—C5—C4	119.6 (8)	N4—C22—C23	113.6 (8)
C6—C5—Br2	121.0 (7)	C28—C23—C24	120.5 (8)
C4—C5—Br2	119.3 (7)	C28—C23—C22	120.2 (7)
C1—C6—C5	122.1 (8)	C24—C23—C22	119.1 (8)
C1—C6—H6	119.0	C25—C24—C23	117.6 (9)
C5—C6—H6	119.0	C25—C24—C30	119.7 (9)
N1—C7—C1	118.5 (7)	C23—C24—C30	122.6 (8)
N1—C7—H7	120.8	C26—C25—C24	121.2 (9)
C1—C7—H7	120.8	C26—C25—H25	119.4
O2—C8—N2	119.5 (7)	C24—C25—H25	119.4
O2—C8—C9	124.9 (9)	C27—C26—C25	121.4 (10)
N2—C8—C9	115.6 (8)	C27—C26—H26	119.3
C14—C9—C10	121.4 (8)	C25—C26—H26	119.3
C14—C9—C8	118.9 (9)	C26—C27—C28	117.7 (9)
C10—C9—C8	119.7 (9)	C26—C27—H27	121.1
C9—C10—C11	116.3 (10)	C28—C27—H27	121.1
C9—C10—C29	123.5 (10)	C23—C28—C27	121.6 (9)
C11—C10—C29	120.1 (11)	C23—C28—H28	119.2
C12—C11—C10	121.4 (11)	C27—C28—H28	119.2
C12—C11—H11	119.3	C10—C29—H29A	109.5
C10—C11—H11	119.3	C10—C29—H29B	109.5
C13—C12—C11	121.6 (11)	H29A—C29—H29B	109.5
C13—C12—H12	119.2	C10—C29—H29C	109.5
C11—C12—H12	119.2	H29A—C29—H29C	109.5
C12—C13—C14	117.5 (10)	H29B—C29—H29C	109.5
C12—C13—H13	121.2	C24—C30—H30A	109.5
C14—C13—H13	121.2	C24—C30—H30B	109.5
C9—C14—C13	121.7 (9)	H30A—C30—H30B	109.5
C9—C14—H14	119.1	C24—C30—H30C	109.5
C13—C14—H14	119.1	H30A—C30—H30C	109.5
C20—C15—C16	120.0 (7)	H30B—C30—H30C	109.5

### Hydrogen-bond geometry (Å, °)

**Table:**

*D*—H···*A*	*D*—H	H···*A*	*D*···*A*	*D*—H···*A*
N2—H2···O4^i^	0.90 (1)	1.85 (2)	2.743 (8)	172 (7)
N4—H4···O2	0.90 (1)	1.92 (2)	2.815 (8)	174 (7)
O3—H3···N3	0.82	1.90	2.624 (8)	146
O1—H1···N1	0.82	1.87	2.584 (8)	145

Symmetry codes: (i) *x*, *y*−1, *z*.
